# Supplementary material for: Mobilisation towards formal employment in the healthcare system: A qualitative study of community health workers in South Africa
Source: PLOS Glob Public Health. 2024 Mar 20;4(3):e0002226. doi: 10.1371/journal.pgph.0002226 (PMC10954165; doi:10.1371/journal.pgph.0002226)
Supplement: Attachment — Submitted filename: PGPH-D-23-01313_Reviewer Comments_(August 2023).docx [file pgph.0002226.s002.docx]

PGPH-D-23-01313

# Abstract

1. The methods sentences can use some detail thus:
   a. Include sample size for each method
   b. add a statement on analysis
2. The results part lacks explicit statements that align with the stated objectives “employment status, struggle for recognition and activities to establish labour representation” to tie down the stated findings. For example, what is the interpretation of the varying CHWs status in rural versus urban?
3. Strengthen the concluding statement.

# Introduction

4. First paragraph reads too general and detached from the manuscript’s discourse, consider starting strongly with the problem which led to your study then narrowing down to what we know about the problem down to what is missing in what we know.
5. Align background aim with the abstract aim.

# Methods

6. Consider adhering to some of the 21 Standards for Reporting Qualitative Research (SRQR).
7. Research design:
   a. State the technical approach utilized in the study and justify its suitability to the study.
8. Study setting:
   a. Consider using maximum variation technique as the criteria for selecting study setting. Add references to the study setting statements.
9. Sampling strategy: Consider having a separate section for sampling embedding it in the data collection section takes away from appreciating the sample population and technique.
10. Data analysis:
    a. State data sources analyzed using thematic content analysis.
    b. Consider stating how authors were involved in the process using their initials.

# Results

11. The first section of the results:
    a. lacks a general statement on the condition (or “status”) of the CHWs thus add as a sweeping statement in response to your initial objective.
    b. Add an interpretative touch to your findings to ease the story articulation. For instance on the issue of career advancement the interpretation may read like “there is no advancement despite finishing training and being qualified”.
    c. The impact of the conditions on effectiveness presented as they are seem as if they are diverting from the study focus on mobilization towards employment; consider framing them in terms of your other objective as part of activities to push change or the struggle itself.
    d. Subthemes lack a storyline, revise to suit the broken-down aim in abstract.
12. Add an interpretive storyline to section two and link to gaps stipulated in background.

# Discussion

13. First paragraph discussion seem like an expansion of results as it lacks references from other sources and comparison to other studies.
14. The discussion section need to be structured in short paragraphs and based on results. Considering the number of subthemes in the results section, the current three paragraphs do not add up.

# Discussion

15. Add recommendations to the conclusion section.
