# Supplementary material for: Mobilisation towards formal employment in the healthcare system: A qualitative study of community health workers in South Africa
Source: PLOS Glob Public Health. 2024 Mar 20;4(3):e0002226. doi: 10.1371/journal.pgph.0002226 (PMC10954165; doi:10.1371/journal.pgph.0002226)
Supplement: Attachment — Submitted filename: Response to reviewers comments 29 11 2023.docx [file pgph.0002226.s003.docx]

Dear Professor Ejemai Eboreime,

PLOS Global Public Health

**RE: Response to Editor’s and Reviewers’ Comments**

Thank you for the feedback dated 15 August 2023. We found the feedback helpful in strengthening the manuscript. We agreed with most of the reviewers' suggestions, in instances where we don’t agree with the reviewers' comments or suggestions we provided a detailed explanation.

**We also want to mention we have decided against using the health districts' real names due to some contextual information being reported in the findings. We have renamed the 3 districts as Districts A, B, C and D, respectively.**

| **Editor’s Comments** | | **Authors responses** |
| --- | --- | --- |
| 1 | The brief background on unions in South Africa is helpful for framing the later findings on CHW unionization  attempts. The one area that could be  strengthened is explicitly stating the study aim/objectives at the end of the introduction section. Currently, the objectives are implicit but not directly stated | Thank you for this feedback.  The study aim and objective is embedded at the end of the introduction. We have revised it and it currently reads as follows:  “In this article, we examine the CHWs’ conditions of employment, their struggle for recognition as health workers, and their activities to establish labour representation in South Africa.” |
| 2 | Some parts of the methods could be  clarified or expanded. In particular, more details on the data analysis process, how themes were derived, and steps to ensure rigour would further strengthen this section.  For the data analysis, consider expanding on the coding process and how themes were derived from the codes. Did you use any specific qualitative analysis approach? How was rigour ensured (e.g., multiple coders, checking themes against raw data)? | As per the suggestion, the data analysis section has also been edited to read as follows:  “As described by Braun [14], we used the thematic analysis method which was inductive to analyse the individual interviews, focus group and participant observation data. The team read through the interview transcripts and observation notes and developed a codebook. The first author HM coded the interview transcripts and observation notes line-by-line, and to ensure the rigor of the findings the coded data and emerging themes were presented to the co-authors (JG and FG). The co-authors verified the coded data against the transcripts. The codes and supporting quotations captured information about the CHWs' conditions of employment and activism to challenge the government to provide permanent employment with decent remuneration (Table 3).” |
| 4 | We have noticed that you have uploaded Supporting Information files, but you have not included a list of legends. Please add a full list of legends for your Supporting Information files after the references list. | We have added a list of legends after the reference list. |
|  | **Reviewer 1** | **Authors responses** |
| 5 | **Methods** a. The selection criteria for the participants for the individual interviews were not clear, especially for CHWs who were also the participants of the focus groups.  b. The distribution of the interviews among the different groups (e.g. CHWs, supervisors, facility staff, etc) was not indicated.  c. A more detailed description on how and where the observations were performed (e.g. health district) would add clarity. | Thank you for the feedback on the methods. We have reviewed the sections and added the details as per the suggestions.   a) the selection criteria have been included in the text:   “In each PHC facility, we interviewed CHWs and their supervisors, facility staff members and nurse mentor (Tables 1). The interviews happened after the observations of the CHW meetings, in order to get clarity on the CHWs' activities. We asked these participants about the CHW programme (i.e. CHWs duties in households, access to resources and supervision, and conditions of employment). Key informants at provincial and district levels were also interviewed.”   b) Table 1 reflects the number of participants per category and facility. c) Description and the location of the observation have been added. The text currently reads as follows:   “The observations were conducted to understand the CHW activities relating to labour mobilisation aimed at challenging the employer to provide better conditions of employment. With the consent of the CHWs, we observed 6 CHW representatives' meetings with the general CHW population, programme coordinators and the payroll administration company in District A (Table 1). In these meetings, we documented the issues being discussed (e.g. poor pay and lack of working tools) and resolutions made.” |
| 6 | **Findings** a. The planned analysis included a comparative analysis across "the different cases of context, processes and outcomes of mobilization", however, these comparisons were not immediately evident in the findings. Perhaps presenting the results across cases in a table would demonstrate similarities and differences across the cases for both CHW employment conditions and CHW mobilization efforts (presented as Table 2) more clearly. | Thank you for this valuable contribution. Table 2 provides a summary of the different cases (e.g. description of the programme, unionisation/mobilisation and associated outcomes).  However, we are aware the table is presented late in the paper. We have decided to move the table forward so the reader can easily scan through the different cases, and mobilisation activities and outcomes. We have also strengthened the table by adding text relating to CHWs and access to resources in the different sites. |
| 7 | b. Please review total number of focus groups in Table 1. (Row 1 - total indicated is 9 but count is 10). | Thank you for pointing this error out. The correct number is 10. |
| 8 | c. If possible, please include a description of the characteristics of the study participants, e.g. for CHWs, age, length of service; for key informants, specify who they were (e.g. supervisor, staff, etc). | This is a good suggestion.  We have included participants' demographics in the findings section. The text read as follows:  “We studied 7 CHW teams in the health districts of Mpumalanga and Gauteng provinces. The teams composition ranged from 8 to 25 CHWs per team. The youngest CHW was aged 26 while the oldest was 61 years old. The CHWs also had varying years of service ranging from 3 years to 17 years. The CHW teams had different supervision configurations consisting of professional nurses and enrolled nurses. The CHWs in Districts A and C were being supervised by professional nurses and enrolled nurses. This was not the case in District D, where the CHWs were being supervised by professional nurses, while the team in District B was being supervised by enrolled nurses. The supervisors were a diverse group aged between 29 years to 65 years. They had worked in different health institutions for 4 to 39 years before joining the government CHW programme. The key informants occupied positions such as programme coordinator and researcher in government departments and universities.” |
| 9 | d. I got the impression that the findings for part 2 on CHW mobilization came from only one health district, Sedibeng. If this is the case, then I suggest that this be stated explicitly in the findings.  Also suggest to specify which interviewer group reported events/findings, i.e. CHW representatives, CHWs, supervisor, etc. | This is true.  We were in the Sedibeng (District A) district for more than 2 years. As a result of our prolonged stay, we had enough time to document the CHWs employment conditions, and engage their task team and union to understand their activities to challenge the government to provide permanent employment with decent pay. We couldn’t do the same for the other sites (i.e. Ehlanzeni, Tshwane and Johannesburg districts) as we weren’t funded. This has now been acknowledged in the methods (study site).  Study site  “The research was carried out in 3 health districts of Gauteng Province and 1 health district of Mpumalanga Province (Table 1). We applied the maximum variation technique to select the two provinces based on their location (urban vs rural), history and contrasting models of implementing the CHW programmes. The CHW programmes in the three districts of Gauteng province were implemented by the Provincial Department of Health with a payroll administration company contracted to pay the CHWs on behalf of the Department [19]. The Mpumalanga province, where the rural case studies were located, had not fully implemented the national CHW programme [19]. Here, the Provincial Department of Health funded existing local NGOs that provided home-based care services, to deploy some of its carers to constitute the CHW programme in local health facilities [19]. The NGOs remained the primary employers of the CHWs, while supervision and resources were being provided by the health facilities [19]. Though we had an interest in the two provinces, we spent 3 years in one district of Gauteng province which were funded. In the other districts, we couldn’t stay long due to limited resources. “  We have also captured this as a limitation of our study.  Discussion  “The study had several strengths and limitations. We spent 3 years collecting data in District A which allowed us to familiarise ourselves with the history of the programme and conflicts between the CHWs and the Department of Health in the district. In the other districts, data collection was limited to three months. Due to our prolonged stay in District A, familiarity with the CHW programme and labour mobilisation, we used the site as the main comparator to the other sites.”  Throughout the findings section, the authors specify which interviewer group reported the findings or events (e.g. CHW, Interview, District B, Team 1). |
| 10 | **Discussion:**  The discussion focused mainly on developments in CHW actions during the COVID-19 pandemic, which, although important, was a different context from what was reported in the findings of the study. A broader discussion of the actions taken by CHWs to push for recognition of their integral role in the healthcare system and factors that have contributed to successful mobilizations/campaigns for better work conditions would have been appreciated. | Thank you for this observation.  In preparing this manuscript, we battled to find papers on CHW mobilisation. We searched databases such as PUBMED and Google Scholar for relevant papers. In these databases, we could only find papers published just before the COVID-19 pandemic and during the pandemic. These papers are cited extensively in the discussion.  However, to accommodate this suggestion, we have strengthened a section in the discussion which highlights some of the mobilisation activities that happened in South Africa prior to the pandemic. The text read as follows:  “Since the end of data collection, South African CHWs have engaged in further industrial action. In 2019, CHWs based in Gauteng province led a protest against the provincial department of health where they were demanding to be absorbed as permanent employees [15]. In 2021, the NEHAWU took the Department of Health to labour court to declare the 12 months contracts illegal and in contravention of the national labour laws, which state employment cannot be offered on a renewable basis for a period exceeding 3 months [19, 20]. The court arbitrator ruled against the union citing a 2018 agreement signed by government and unions. The agreement refers to government dependence on an external grant to pay CHW current salaries, such that CHWs permanent employment is unsustainable.” |
|  | **Reviewer 2** | **Authors responses** |
| 11 | **Abstract**  1. The methods sentences can use some detail thus: a. Include sample size for each method b. add a statement on analysis  2. The results part lacks explicit statements that align with the stated objectives “employment status, struggle for recognition and activities to establish labour representation” to tie down the stated findings. For example, what is the interpretation of the varying CHWs status in rural versus urban?  3. Strengthen the concluding statement. | The abstract has been reviewed. Details such as the sample size per method of data collection and analysis method have been added. It currently reads as follows:  “In low and middle-income countries (LMICs), community health workers (CHWs) play a critical role in delivering primary health care (PHC) services to vulnerable populations. In these settings, they often receive low stipends, function with a lack of basic resources and have little bargaining power with which to demand better working conditions. In this article, we examine CHWs’ employment status, their struggle for recognition as health workers, and their activities to establish labour representation in South Africa. Using a qualitative case study methodology, we studied seven CHW teams located in semi-urban and rural areas of Gauteng and Mpumalanga Provinces, South Africa. We conducted 43 in-depth interviews, 10 focus group discussions and 6 observations to gather data from CHWs and their representatives, supervisors and PHC facility staff members. The data was analysed using the thematic analysis method. In the rural and semi-urban sites, the CHWs were found to be poorly supervised, resourced and received meagre remuneration, their employment outsourced, without employment benefits and protection. The lack of career progression opportunities demotivated the CHWs, particularly those keen to establish a career in health. In the semi-urban sites, CHWs established a task team to represent them that held regular meetings and often used violent and disruptive strategies against clinics, district and provincial management, which often led to tensions and conflicts with facility staff and programme coordinators. After a meeting with the local provincial legislature, the task team joined a labour union (NEHAWU) in order to be able to participate in the local Bargaining Council. Though they were not successful in getting the government to provide permanent employment, the union negotiated an increase in stipend from R2 500 (136 USD) to R3 500 (192 USD). In contrast, in the rural sites, the CHWs were not actively demanding permanent employment due to their employment contracts being partly managed by non-government organisations (NGOs); they were fearful of being removed from the government programme. After the study ended, during the height of COVID-19 in 2020, when the need for motivated and effective CHWs became much more obvious to decision makers, the semi-urban-based teams received permanent employment with remuneration between R9-11,000 (500-600 USD). The task team and their protests raised awareness of the plight of the CHWs, and joining a formal union enabled them to negotiate a modest salary increase. However, it was the emergency created by the worldwide COVID-19 pandemic that forced decision-makers to acknowledge their reliance on this community-based cadre. Hopefully, this recognition, and the associated gains, will not fade as the pandemic recedes.” |
| 12 | **Introduction** 4. First paragraph reads too general and detached from the manuscript’s discourse, consider starting strongly with the problem which led to your study then narrowing down to what we know about the problem down to what is missing in what we know. | Thank you for the suggestion. We revisited the first and second paragraphs. The text now integrate the history of CHW programmes (the Alma Ata declaration of 1978) and CHWs challenges including poor conditions of employment and mobilisation (focus of the study):  “In pursuit of universal health coverage (UHC), low and middle-income countries (LMICs) are investing in programmes to strengthen the delivery of primary health care services (PHC) to marginalised communities [1]. The community health worker (CHW) programmes gained prominence after the Alma Ata Conference of 1978, which prioritised the strengthening of PHC to provide care to marginalised communities [2]. Since this period, many LMICs have been making attempts to use CHWs to provide care [2]. In some settings, the CHWs have been successful in extending access to PHC services by supporting chronic care management, antenatal and postnatal support and early identification of malnourished children [2-4].  However, in some settings, the programmes fail to deliver on expectations due to limited access to resources (e.g. blood pressure and glucose monitoring machines), low and sometimes irregular remuneration and poor integration into the healthcare system resulting in poor relationships with facility-based staff members [3, 5, 6]. CHWs are mostly women, often with low levels of education, who are expected to work on a voluntary basis, or who are underpaid with no permanent employment security [7]. These employment conditions and the lack of unionisation contribute to CHWs’ low work morale, demotivation and high work turnover [1, 8]. Pandya et al. argues the provision of incentives, both monetary and non-monetary is crucial to improve CHW motivation, job satisfaction, and performance, which, in turn, improves retention [7]. Monetary incentives include stable salaries, while non-monetary incentives include the provision of working tools such as uniforms and raincoats.” |
| 13 | 5. Align background aim with the abstract aim. | The abstract and introduction/aim text have been reviewed and aligned.  “In this article, we examine the CHWs’ employment conditions, their struggle for recognition as health workers, and their activities to establish labour representation in South Africa.” |
| 14 | **Methods**  Consider adhering to some of the 21 Standards for Reporting Qualitative Research (SRQR). | We have included the SRQR checklist. The manuscript adheres to the 21 checklist. Reference to specific text speaking to each domain/checklist is indicated in the table. |
| 15 | **Research design:** a. State the technical approach utilized in the study and justify its suitability to the study. | The research followed the qualitative approach. A definition of qualitative approach has been provided and justified. The text read as follows:  “The two studies adopted a qualitative approach with 7 case studies (each case study being a team of CHWs). Creswell and Clark define qualitative research as an approach used by researchers to gain an in-depth understanding of a particular phenomenon [18]. Through this approach, the researchers undertook an in-depth exploration of the CHWs' conditions of employment and mobilisation activities for recognition in the healthcare system.” |
| 16 | **Study setting:** a. Consider using maximum variation technique as the criteria for selecting study setting.  Add references to the study setting statements. | Thank you for this suggestion. The below text has been reordered to clearly specify the use of this technique and reasons.  “The research was carried out in 3 health districts of Gauteng Province and 1 health district of Mpumalanga Province (Table 1). We applied the maximum variation technique to select the two provinces based on their location (urban vs rural), history and contrasting models of implementing the CHW programmes. The CHW programmes in the three districts of Gauteng province were implemented by the Provincial Department of Health with a payroll administration company contracted to pay the CHWs on behalf of the department [19]. The Mpumalanga province, where the rural case studies were located, had not fully implemented the national CHW programme [19]. Here, the Provincial Department of Health funded existing local NGOs that provided home-based care services, to deploy some of its carers to constitute the CHW programme in local health facilities. The NGOs remained the primary employers of the CHWs, while supervision and resources were being provided by the health facilities [19]. Though the researchers had an interest in the two provinces, we spent 3 years in the Sedibeng district sites which were funded. In the other districts, we couldn’t stay long due to limited resources.  We have added the references. |
| 17 | **Sampling strategy:** Consider having a separate section for sampling. Embedding it in the data collection section takes away from appreciating the sample population and technique. | Thank you for this suggestion.  We have added “sampling and participant recruitment” . This subsection highlight the sampling techniques employed and read as follows:  “We employed a combination of purposive and snowball sampling techniques to select the participants for the study [20]. All CHWs and supervisors who formed part of the CHW programmes and selected facility staff members in the PHC facilities were purposively invited to participate in the study. The snowball sampling technique was used to select key informants in government and universities in Gauteng and Mpumalanga provinces (Table 2). The informants’ inclusion criteria were a sustained knowledge of CHW programmes, and involvement in the set-up, implementation and evaluation of the programmes performance in the different sites.” |
| 18 | **Data analysis:** a. State data sources analyzed using thematic content analysis. b. Consider stating how authors were involved in the process using their initials. | We analysed the individual interviews, focus group discussions and observations using thematic analysis. This information as well as the authors contributions in the analysis process is now reflected in the text below:  “As described by Braun [14], we used the thematic analysis method which was inductive to analyse the individual interviews, focus group and participant observation data. The team read through the interview transcripts and observation notes and developed a codebook. The first author HM coded the interview transcripts and observation notes line-by-line, and to ensure the rigor of the findings the coded data and emerging themes were presented to the co-authors (JG and FG). The co-authors verified the coded data against the transcripts. The codes and supporting quotations captured information about the CHWs' conditions of employment and activism to challenge the government to provide permanent employment with decent remuneration.” |
| 19 | **Results** The first section of the results: a. lacks a general statement on the condition (or “status”) of the CHWs thus add as a sweeping statement in response to your initial objective. | The text has been reviewed and currently read as follows:  “The findings comprise participant demographics, CHWs conditions of employment (i.e. stipend, employment security, career advancement opportunities, work tools and access to supervision), and how these conditions hindered the effectiveness of the CHWs in the different sites. Lastly, the CHW labour mobilisation towards permanent employment with decent remuneration is presented.”  We would prefer not making a sweeping statement about our results at the beginning of this section. To our mind it is better to present our data, and then at the beginning of the discussion, summarise our findings, drawing out the implications. In this order, the reader is in a position to judge whether our summary, and our assessment of the implications is actually valid or not. We hope you agree with us. |
| 20 | b. Add an interpretative touch to your findings to ease the story articulation. For instance on the issue of career advancement the interpretation may read like “there is no advancement despite finishing training and being qualified”. | This is a good suggestion. I have reviewed the findings section, reordered the text and added interpretation to most of the themes. Examples:  “**Career advancement opportunities**  A representative of the CHWs in Sedibeng was concerned that the Department of Health provides short training courses but never considers them (CHWs) for promotion after the training: “*After some of them finished and got their certificates; they are still doing the same work, same level, same stipend. So, that is our biggest challenge. Why are there trainings that don’t have opportunities?*” (CHW representative, Interview 1, District A). This view was shared by the CHWs: “*We complete trainings. At least when you have done the training, they should promote you but no we remain in the same posts.*” (CHW, FGD, District B). It appears there was no career advancement for the CHWs even after completing training.  **Resources**  When undertaking household visits, CHWs require resources such as blood pressure and glucose monitoring machines, stationary and a raincoat etc. At the inception of the programme, the Department of Health made provisions for the CHWs to have these resources, although some teams did not receive them: “*We only saw the boxes. One sister from clinic X did ask how come we don’t have BP machines. We said we don’t know. She promised to get us BP machines. We got 2 machines and we had to share them but now there are no batteries*.” (CHW, FGD, District D, Team 2). During data collection, the majority of the CHWs still did not have these resources (Table 3).” |
| 21 | c. The impact of the conditions on effectiveness presented as they are, seem as if they are diverting from the study focus on mobilization towards employment; consider framing them in terms of your other objective as part of activities to push change or the struggle itself. | The study set out to explore the CHW conditions of employment and mobilisation (e.g. joining a labour union) to negotiate for permanent employment and access to resources.  To ensure alignment of information, the study aim has been clarified in the abstract and introduction. Furthermore, the findings have also been reviewed in line with suggestion number 20 to clearly highlight the CHWs frustrations which led to mobilisations. |
| 22 | d. Subthemes lack a storyline, revise to suit the broken-down aim in abstract. | The findings section has been revised to be in line with the abstract and introduction section. Some of the themes were rewritten to better fit the text. |
| 23 | Add an interpretive storyline to section two and link to gaps stipulated in background. | This is a good suggestion. We have added the below section:  “As described above, The CHWs were frustrated by the lack of working tools, poor remuneration and absence of career advancement opportunities. In this section, we highlight the CHWs' activities to challenge the government to improve their conditions of employment.” |
| 24 | **Discussion** First paragraph discussion seem like an expansion of results as it lacks references from other sources and comparison to other studies. | Thank you for the feedback on the discussion.  As mentioned above, we have used the first paragraph to draw out and summarise the key findings, as we find it helps as a starting point for the discussion. As a result, it doesn’t have any references.  In the main body of the discussion, we have picked on the key issues that have implications for policy and practice, and further research, to discuss them in greater depth. We have ensured that each paragraph starts with a topic sentence to help indicate to the reader the focus of each paragraph, and how the section is structured.  We have also strengthened the discussion by adding two paragraphs focused on CHWs lack of resources and need for career advancement opportunities. Below, we explain the focus of each paragraph.  Paragraph 1: provide a summary of the key findings  Paragraph 2: describes CHWs lack of crucial resources and impact on services  Paragraph 3: Describes the need for countries to put in place measures to upskill and develop CHWs through career advancement opportunities.  Paragraph 4 and 5: describes CHWs' efforts for recognition prior to and during the height of the COVID-19 pandemic.  Paragraph 6: reinforces the lack of unionisation in the CHW sector and how different CHWs are forming unions.  Paragraph 6: highlight the study's strengths and weaknesses.  We trust you find this in order. |
| 25 | The discussion section need to be structured in short paragraphs and based on results. Considering the number of subthemes in the results section, the current three paragraphs do not add up. | Kindly note our response in point 24, where we explain the structure of the discussion and the changes introduced to strengthen the section. |
| 26 | Add recommendations to the conclusion section. | This is a good suggestion. We have incorporated some recommendations into the conclusion. See below:  “Consistent mobilisation as demonstrated by the urban-based teams enabled the CHWs to successfully negotiate salary increments and advance their call for permanent employment. However, in the rural sites, the CHWs were less able to join or establish labour representation due to fear of reprisal from NGOs management. It is important that CHWs are afforded the right to belong to a labour union in order to be able to negotiate for decent employment conditions. In optimising the motivation and performance of CHWs, the government needs to prioritise the full integration of CHWs into the healthcare system, where they will be afforded their labour rights and support. This could include a provision of decent salary with benefits such as leave and career progression opportunities for interested CHWs.” |

**Kindest Regards**

Hlologelo Malatji on behalf of the authors

Centre for Health Policy, South Africa
