# Supplementary material for: Mobilisation towards formal employment in the healthcare system: A qualitative study of community health workers in South Africa
Source: PLOS Glob Public Health. 2024 Mar 20;4(3):e0002226. doi: 10.1371/journal.pgph.0002226 (PMC10954165; doi:10.1371/journal.pgph.0002226)
Supplement: Attachment — Submitted filename: PGPH-D-23-01313_Reviewer Comments_(December 2023).docx [file pgph.0002226.s004.docx]

# PGPH-D-23-01313R1

# Abstract

1. The methods section has been revised to include sample size and data analysis strategy.
2. The results section now aligns with the stated objectives “employment status, struggle for recognition and activities to establish labor representation”. However they need to be framed coherently;
   - for instance, line 26 to 28 is a list of challenges which are expected to be unpacked as a trigger to protests but the sentence that follows also presents another trigger then jumps to industrial action aimed at improving employment condition “permanent employment and pay increment” which only appear in next sentence.
3. The benefits seem to be for semi-urban only, what of the rural? Include their fate to balance the conclusion.

# Introduction

4. The background’s aim now aligns with the abstract’s aim.

# Methods

5. The methods section has been well revised, it now meets acceptable standards for reporting qualitative research.

# Results

6. The results have been improved but lack the aftermath version presented in the abstract. Decide whether you add document review (how you got the semi-urban news of improvements) as a new method to have full circle results.

# Discussion

7. The discussion section has been revised to well specified thematic discussion paragraphs.
8. The discussion on the aftermath on line 583 to line 611 seems like a mix of results and discussion. It also lacks statements of other settings “Mpumalanga”, be it there is no information or such changes are yet to take effect.

# Conclusion and Recommendations

9. A conclusion and recommendations have been added. Any recommendation for further study?
