# Supplementary material for: Mobilisation towards formal employment in the healthcare system: A qualitative study of community health workers in South Africa
Source: PLOS Glob Public Health. 2024 Mar 20;4(3):e0002226. doi: 10.1371/journal.pgph.0002226 (PMC10954165; doi:10.1371/journal.pgph.0002226)
Supplement: Attachment — Submitted filename: Response to reviewers comments 15 1 2024.docx [file pgph.0002226.s005.docx]

Dear Professor Ejemai Eboreime,

PLOS Global Public Health

**RE: Response to Editor’s and Reviewer’s Comments**

Thank you for the feedback dated 29 December 2023. We found the feedback helpful in strengthening the manuscript. We agreed with most of the reviewers' suggestions, in instances where we don’t agree with the reviewers' comments or suggestions we provided a detailed explanation.

| **Editor’s comments** | **Responses** |
| --- | --- |
| The manuscript is much improved. Please attach a completed COREQ checklist indicating how your manuscript aligns with the reporting standards. | Thank you for the positive feedback. I have attached the COREQ checklist |
|  |  |
| **Reviewer’s comments** | **Responses** |
| **Abstract**  The methods section has been revised to include sample size and data analysis strategy. | Thank you for the feedback |
| The results section now aligns with the stated objectives “employment status, struggle for recognition and activities to establish labor representation”. However they need to be framed coherently;  For instance, line 26 to 28 is a list of challenges which are expected to be unpacked as a trigger to protests but the sentence that follows also presents another trigger then jumps to industrial action aimed at improving employment condition “permanent employment and pay increment” which only appear in next sentence. | Thank you for pointing this gap out. We have carefully went through the sections and realist it was unclear how the protests started and what was achieved.  Minor amendment have been made to clearly highlight the protests were first forged by the CHWs and task team (CHWs make the task team) and when not successful in gaining the attention of the government, they joined a formal union to represent them. It is only through the union that they achieved salary increment.  “In low and middle-income countries (LMICs), community health workers (CHWs) play a critical role in delivering primary health care (PHC) services to vulnerable populations. In these settings, they often receive low stipends, function without resources and have little bargaining power with which to demand better working conditions. In this article, we examine CHWs’ conditions of employment, their struggle for recognition as health workers, and their activities to establish labour representation in South Africa. Using a qualitative case study methodology, we studied seven CHW teams located in semi-urban and rural areas of Gauteng and Mpumalanga Provinces, South Africa. We conducted 43 in-depth interviews, 10 focus group discussions and 6 observations to gather data from CHWs and their representatives, supervisors and PHC facility staff members. The data was analysed using thematic analysis method. In addition, we carried out document a review which included government and media articles analysis to understand the CHWs conditions of employment post the research. In the rural and semi-urban sites, the CHWs were found to be poorly supervised, resourced and received meagre remuneration, their employment outsourced, without employment benefits and protection. The lack of career progression opportunities demotivated the CHWs, particularly those keen to establish a career in health. As a result of these challenges, the CHWs in the semi urban sites established a task team to represent them that held regular meetings and often used disruptive strategies against the clinics, district and provincial management, which often led to tensions and conflicts with facility staff and programme coordinators. After numerous unsuccessful attempts to negotiate for improved conditions of employment on their own, the task team joined a labour union (NEHAWU) in order to be able to participate in the local Bargaining Council. Though they were not successful in getting the government to provide permanent employment, the union negotiated an increase in stipend from R2 500 (136 USD) to R3 500 (192 USD). In contrast, in the rural sites, there were less CHWs mobilisations and unionisation. The CHWs were not actively demanding permanent employment due to their employment contracts being partly managed by non-government organisations (NGOs); they were fearful of being removed from the government programme. After the study ended, during the height of COVID-19 in 2020, when the need for motivated and effective CHWs became much more obvious to decision makers, the semi-urban-based teams received permanent employment with remuneration of between R9-11,000 (500-600 USD). The task team and their protests raised awareness of the plight of the CHWs, and joining a formal union enabled them to negotiate a modest salary increase. However, it was the emergency created by the world-wide COVID-19 pandemic that forced decision-makers to acknowledge their reliance on this community-based cadre. Hopefully, this recognition, and the associated gains, will not fade as the pandemic recedes. “ |
| The benefits seem to be for semi-urban only, what of the rural? Include their fate to balance the conclusion. | Thank you for pointing this out. We have now added text in the abstract that mention there were less mobilisation and unionisation to demand better employment conditions in the rural areas. |
| **Results**  The results have been improved but lack the aftermath version presented in the abstract. Decide whether you add document review (how you got the semi-urban news of improvements) as a new method to have full circle results**.** | Thank you.  We agree to include document review is one of the methods we used to gather data. Document review is now mentioned in the abstract and methods (table).  The results now incorporate text that that highlight the gains observed post data collection and during the height of the COVID-19 pandemic. The text read as follows:  “Post the study and during the height of COVID-19 in 2020, when the need for motivated and effective CHWs became much more obvious to decision makers, the semi-urban-based teams received permanent employment with remuneration of between R9-11,000 (500-600 USD). The other CHW teams located in the rural areas did not receive this increment**.”** |
| **Discussion**  The discussion section has been revised to well specified thematic discussion paragraphs. | Thank you |
| The discussion on the aftermath on line 583 to line 611 seems like a mix of results and discussion. It also lacks statements of other settings “Mpumalanga”, be it there is no information or such changes are yet to take effect. | The discussions has been looked into. We have reworded some of the sections to differentiate our findings and literature.  Text highlighting the differences between the two provinces (Gauteng and Mpumalanga provinces) has been added. |
| **Conclusion and recommendations**  A conclusion and recommendations have been added. Any recommendation for further study? | Thank you.  We have added a recommendation for further study  “Consistent mobilisation as demonstrated by the urban-based teams enabled the CHWs to successfully negotiate salary increments and advance their call for permanent employment. However, in the rural sites, the CHWs were less able to join or establish labour representation due to fear of reprisal from NGOs management. It is important that CHWs are afforded the right to belong to a labour union in order to be able to negotiate for decent employment conditions. In optimising the motivation and performance of CHWs, the government need to prioritise the full integration of CHWs into the healthcare system, where they will be afforded their labour rights and support. This could include a provision of a decent salary with benefits such as leave and career progression opportunities for interested CHWs. It is further recommended that future studies prioritise the exploration of the CHWs conditions of employment post the COVID-19 pandemic. As previously stated, the pandemic highlighted the important role CHWs play in delivering healthcare to marginalised communities. Therefore it is important to assess whether their conditions have changed since the pandemic. “ |

**Kindest Regards**

Hlologelo Malatji on behalf of the authors

Centre for Health Policy, South Africa
